# Prompting social investigation

**DOI:** 10.7554/eLife.99363

**Published:** 2024-06-12

**Authors:** Emma Keppler, Susanna Molas

**Affiliations:** 1 Institute for Behavioral Genetics, University of Colorado Boulder Boulder United States; 2 Department of Psychology and Neuroscience, University of Colorado Boulder Boulder United States; 3 Crnic Institute Boulder Branch, BioFrontiers Institute, University of Colorado Boulder Boulder United States

**Keywords:** behavior, social investigation, ventral hippocampus, lateral septum, ventral tegmental area, Mouse

## Abstract

A social memory pathway connecting the ventral hippocampus, the lateral septum and the ventral tegmental area helps to regulate how mice react to unknown individuals.

**Related research article** Rashid M, Thomas S, Isaac J, Karkare S, Klein H, Murugan M. 2024. A ventral hippocampal-lateral septum pathway regulates social novelty preference. *eLife* **13**:RP97259. doi: 10.7554/eLife.97259.

Animals often need to assess whether a member of their species (a conspecific) that they have not met before will be a friend or a foe. As such, most adult animals would tend to investigate an unfamiliar peer over one which they were already acquainted with (Tapper and Molas, 2020).

Deciding whether and how to engage with an unknown individual relies on multiple levels of analysis underpinned by different brain networks and areas. First, the animal must identify that it has not encountered this specific peer before. For this, it must detect and check discrete features in the new conspecific against information deposited in memory networks after previous encounters. Certain regions of the hippocampus (the brain structure that helps to form memory and process emotions) have been implicated in this mechanism. Hippocampal neurons in the CA2 region and in the ventral portion of the CA1 area, for example, store social memories that allow animals to distinguish between new and familiar conspecifics (Hitti and Siegelbaum, 2014; Okuyama et al., 2016).

Once an unknown conspecific has been identified, other brain areas are then required to determine the appropriate course of action — whether to approach or retreat, for instance — and to prompt the associated behaviors. Emerging evidence indicates that the lateral septum may be involved in this process (Menon et al., 2022). This brain area – which is mostly formed of inhibitory neurons that repress the activity of the cells they project onto – is known to help shape social and emotional behaviors. The lateral septum receives projections from both the dorsal and ventral segments of the hippocampus and, in turn, connects with various regions involved in goal-directed behaviors. This includes the ventral tegmental area (or VTA; Rizzi-Wise and Wang, 2021). When dopaminergic neurons in this part of the brain are activated, such as during novel social interactions, they help drive the exploration of new stimuli and conspecifics (Gunaydin et al., 2014; Solié et al., 2022; Molas et al., 2024). Yet many of these pathways remain poorly understood. In particular, it is still unclear how the ventral hippocampus interacts with the lateral septum and the VTA to ‘transform’ social memories into motivations that promote individuals to investigate new conspecifics.

Now, in eLife, Malavika Murugan and colleagues at Emory University – including Maha Rashid as first author – report a new pathway between the ventral hippocampus, the lateral septum, and the VTA that regulates social novelty preference in mice (Figure 1; Rashid et al., 2024). To identify this circuit, the team carried out a social discrimination test which involved placing a mouse in an open chamber alongside two conspecifics of the same age and sex, which were caged on opposite sides of the apparatus. Only one of these individuals was known to the test subject, as they had been housed together for 72hours prior to the experiment. This is a much longer period than used in other protocols, allowing the animals to better recognize the features of the familiar peer.

**Figure 1.**
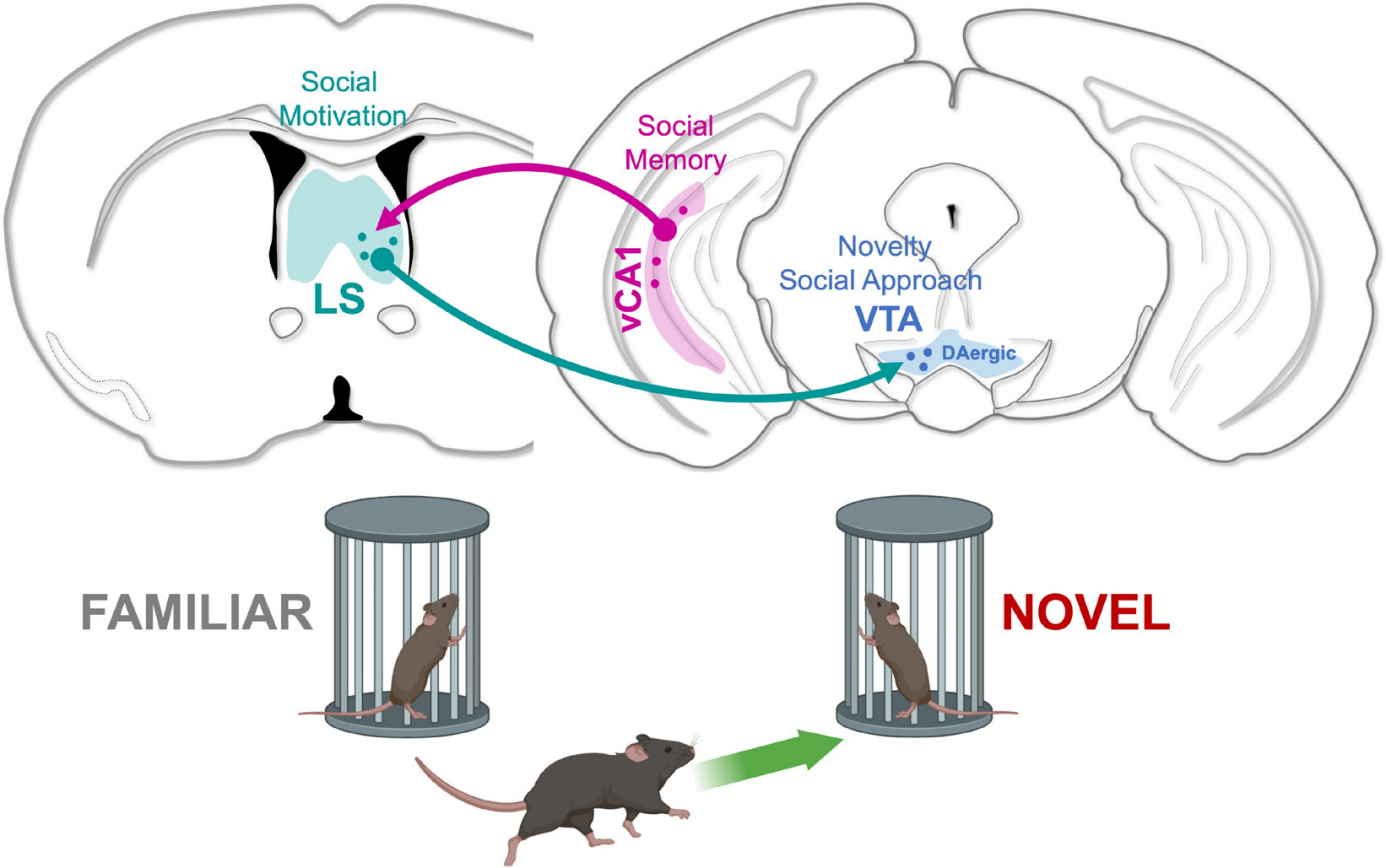
A neuronal circuit controlling social preferences. vCA1 neurons (pink) in the ventral hippocampus encode social information and project to the lateral septum (LS; green), a region involved in social motivation. Lateral septum neurons establish monosynaptic connections onto dopaminergic neurons in the ventral tegmental area (VTA; blue), which promote exploration (green arrow) of new individuals.

Preference for social novelty was determined by the amount of time the subject spent exploring the new individual relative to the familiar one. Without interventions, the mouse spent longer around the new conspecific.

Rashid et al. then used a technique known as chemogenetics to deactivate the neural pathway connecting the ventral hippocampus to the lateral septum, as this allowed them to assess whether these projections are required for animals to discriminate between novel and familiar social stimuli. The treatment did not affect the mice’s tendency to investigate new foods or objects more, but it disrupted their preference for social novelty (that is, the animals spent similar amounts of time investigating unknown and familiar individuals).

The team then used optogenetics to explore this effect in more detail, as this approach makes it possible to temporarily deactivate the pathway ‘at will’ as the animals perform the test. The experiments showed that the mice preferred investigating the conspecific that had been physically closest to them at the time their pathway had been silenced. Switching off the pathway promoted investigative behaviors towards a familiar individual (with the mice then having less time to spend exploring the unknown conspecific). In addition, if exposed to two new peers, the subjects explored the one which had been nearby during the manipulation. Overall, this suggests that preventing the activation of this pathway results in social investigations being more engaging. This led Rashid et al. to propose that the ventral hippocampus-lateral septum pathway may inhibit downstream regions which drive exploration of new social stimuli, such as the VTA.

The team therefore examined next whether the ventral hippocampus projects onto the pathway connecting the lateral septum to the VTA. To do so, they used monosynaptic rabies tracing, a method that helps reveal which neurons directly communicate with a specific cell. This allowed Rashid et al. to establish that the ventral hippocampus innervates cells in the lateral septum which connect to the VTA; disrupting the latter pathway with chemogenetic tools also prevented the preference for a novel mouse. Crucially, rabies tracing allowed Rashid et al. to show that neurons in the lateral septum directly project onto dopaminergic neurons in the VTA. In particular, the rostral part of the lateral septum, a subdivision recently implicated in the shift from novel to familiar social preferences in young mice, projected most strongly to the dopaminergic cells (de León Reyes et al., 2023).

Taken together, these results reveal a pathway connecting social memories stored in the ventral hippocampus to the centers responsible for motivational social behaviors (Figure 1). Ventral hippocampal cells connect to lateral septum neurons that are important for social behavior, which, in turn, project to VTA dopaminergic centers that control the animal’s social approach. Inhibition of the hippocampal-septal pathway disinhibits these VTA centers, resulting in the mouse being more interested in novel social interactions. These findings will aid in developing new therapies that improve social impairments in numerous neurodevelopmental and neuropsychiatric disorders.
